# Combination of Mpox With Ocular Involvement, Human Immunodeficiency Virus, and Pulmonary Tuberculosis Resulting in High Mortality: A Report of Two Cases

**DOI:** 10.7759/cureus.93890

**Published:** 2025-10-05

**Authors:** Yulia Aziza, Golda Asina Miranda Simanjuntak, Lukman Edwar

**Affiliations:** 1 Department of Ophthalmology, Faculty of Medicine, University of Indonesia, Jakarta, IDN; 2 Department of Ophthalmology, Cipto Mangunkusumo Hospital, Jakarta, IDN

**Keywords:** co-infection, hiv, mpox, ocular mpox, tuberculosis

## Abstract

Mpox is a reemerging zoonotic viral disease that has been declared a public health emergency. Ocular symptoms of mpox include pain, redness, tearing, discharge, swelling, and vision impairment. This report describes two cases of mpox with ocular involvement in Indonesia, where the patients had a history of multiple male partners (men who have sex with men (MSM)), human immunodeficiency virus (HIV), and tuberculosis (TB). The first case was a 28-year-old male patient with multiple scabs, including in the eye area, shown as right-eye blepharoconjunctivitis. Despite treatment with tecovirimat, he died from respiratory failure. The second case involved a 25-year-old male patient with similar symptoms and blepharoconjunctivitis in both eyes. He did not receive tecovirimat and also died from respiratory failure. Mpox can severely affect organs, including the eyes. The risk of mpox fatality increases with HIV and TB co-infection. Early recognition of the disease signs may improve the prognosis.

## Introduction

Mpox resurfaced post-COVID-19, mainly in Africa. Initially transmitted from animals in 1958, human-to-human transmission was noted in 1996-1997. Mpox is clinically comparable to smallpox and causes lymphadenopathy, tiredness, and vesico-pustular skin lesions [1,2]. The World Health Organization (WHO) proclaimed mpox a public health emergency in 2022 [1].

While usually self-limiting, mpox can lead to severe complications like myocarditis and encephalitis. Ocular involvement, though rare, may cause vision loss [2-4]. Mpox patients shall undergo screening for sexually transmitted infections (STIs) such as human immunodeficiency virus (HIV), a common co-infection of mpox, and other infectious diseases, including tuberculosis (TB) [1,3]. Mpox, which occurs alongside other infections, might complicate diagnosis, symptoms, and treatment. Studies have reported that coinfections with HIV and other STIs are associated with higher fatality rates, poorer clinical outcomes, and prolonged recovery [5]. This report highlights the high mortality risk of ocular mpox cases in Indonesia, which had a history of multiple male partners (men who have sex with men (MSM)), HIV, and TB.

## Case presentation

This study included subjects with a confirmed polymerase chain reaction (PCR) diagnosis of mpox and coinfections of HIV, TB, or other STIs and had ocular involvement within the year 2023 who were hospitalized at Cipto Mangunkusumo National General Hospital, Jakarta, Indonesia. There were two cases included in the case series. 

Case 1

**Figure 1:**
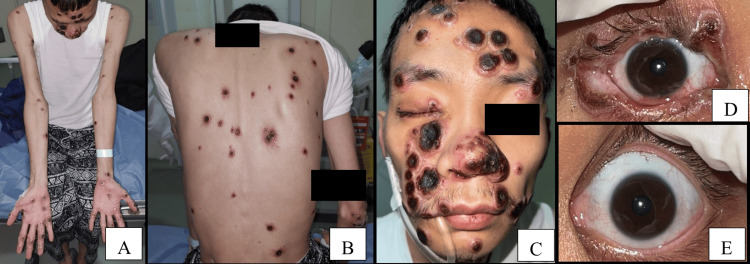
The patient presented with painless black plaque crusts. (A) Upper and lower trunk; (B) back side; (C) face with black plaque crusts; (D) right eye with hyperemic, multiple black lesions, eyelid, and conjunctival injection; (E) normal left eye condition The immediate kin of the patient consented to have the patient's images used in an open-access publication. A written and signed consent statement was provided to the journal.

A 28-year-old male patient with pulmonary TB treated with anti-TB presented with fever, cough, dyspnea, and skin lesions. The lesions, initially whitish boils, had turned into scabs, hardened crusts of blood covering the trunk, limbs, and face (Figures 1A-1C). He reported sexual activity with a male partner, with the last encounter three months prior and no prior mpox vaccination. Mpox was confirmed via PCR using a specimen from skin lesions. During the hospitalization, he was also diagnosed with HIV (CD4 55 cells/mm³) and neurosyphilis. He could not start the antiretroviral therapy (ART) due to elevated liver function tests related to anti-TB. For the neurosyphilis, he received intramuscular benzathine penicillin.

Ocular symptoms included right eye redness. Visual acuity was 6/24 in the right eye and 6/12 in the left. The right eyelid showed multiple scabs on both the upper and lower eyelids, with mild conjunctival injection. The anterior segment of the eye was normal, and the left eye was unaffected (Figures 1D, 1E). He was diagnosed with mpox-induced blepharoconjunctivitis from PCR using specimens from the skin of a periocular lesion. Treatment included levofloxacin, prednisolone acetate, and carboxymethylcellulose sodium eye drops for ocular involvement, and systemic tecovirimat and ART. Despite treatment, his condition worsened, leading to respiratory failure, and he passed away.

Case 2

**Figure 2:**
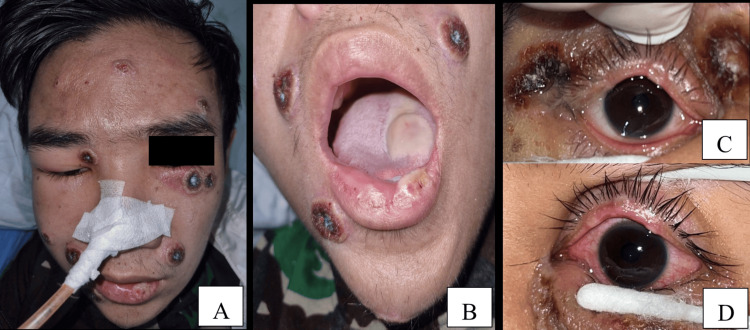
Ocular clinical findings of Case 2 The patient presented with painless, multiple lenticular to nummular, discrete black crusts on the face (A) and mouth (B). The right eye with edematous, erythematous, black, ulcerated crust on the eyelid (C) and the left eye with erythematous eyelid and conjunctival injection (D) were seen. The immediate kin of the patient consented to have the patient's images used in an open-access publication. A written and signed consent statement was provided to the journal.

A 25-year-old male patient diagnosed with HIV four months ago (CD4 6 cells/mm³) and pulmonary TB, who had been on anti-TB treatment for three months, had a history of travelling to Malaysia and presented with vague abdominal pain and scabs distributed all over his body. The lesions spread from his lower trunk to his face, forming black crusts (Figure 2A). Nine days later, new lesions appeared on his right palm, left sole, and lower lip (Figure 2B). He identified as an MSM and reported that he had sexual activity two months ago. Mpox PCR using a specimen collected from the skin lesion confirmed the diagnosis.

Ocular findings (Figures 2C, 2D) included swollen eyelids without visual disturbances. Visual acuity was 6/6 in both eyes. The right eye had reddish edema and a black ulcerated crust, while the left eye showed conjunctival injection but no corneal involvement. He was diagnosed with blepharoconjunctivitis secondary to mpox. Treatment included topical levofloxacin, acyclovir, and artificial tears. His ocular condition improved after three days. Levofloxacin was given to prevent secondary infection.

The patient had not been treated with ART before the hospitalization due to the patient’s personal reasons, and ART was started during his stay. Since systemic tecovirimat was unavailable throughout the country during that time, the patient received oral acyclovir. Two weeks later, he developed acute lung edema and passed away after treatment failed to stabilize his condition.

## Discussion

Mpox, a double-stranded DNA virus from the Poxviridae family, was first transmitted from animals to humans in 1958, with a subsequent outbreak in 1996. MSMs and those who are co-infected with HIV were primarily impacted by the global mpox outbreak in 2022 [1, 3, 4]. The WHO reported more than 92,000 cases as of May 2024, of which 51.9% were HIV-positive and 96.4% were male [1]. Both of the study’s cases, which involved males with HIV and same-sex transmission, are consistent with the worldwide pattern and also had low CD4 counts and had not yet started ART. Severe symptoms of mpox may be found in cases with co-infection with HIV, STIs, or bacterial superinfections [5, 6]. These cases show deteriorated outcomes and contribute to the high mortality of mpox due to co-infection. 

Mpox symptoms are similar to smallpox, but lymphadenopathy and skin lesions that progress to vesicopustular forms often affect the periorbital and anogenital areas [1,4,7]. It is typical for mucosal involvement to occur, including in the eyes. In 2003, the first case of ocular involvement was documented as conjunctivitis and blepharitis [8]. During the 2022 outbreak, eyelid lesions and ocular redness were common ocular symptoms; in certain cases, discomfort, photophobia, and preauricular lymphadenopathy were also observed [1, 7, 9]. Patients exhibited eyelid lesions and conjunctival injection but no corneal involvement, consistent with prior findings.

There are no standard treatment guidelines for ocular mpox. Vaccinia vaccine within four days to two weeks of exposure is recommended by the Centers for Disease Control and Prevention (CDC) [1, 10, 11]. Tecovirimat, an anti-poxvirus agent, is the preferred treatment, but alternative therapies like brincidofovir and cidofovir are also used, despite serious side effects. Trifluridine eye drops are suggested for ocular mpox but are not available in Indonesia [2, 12]. Our first patient received tecovirimat, while the second patient received acyclovir due to tecovirimat’s unavailability, resulting in similar mortality due to case complexity. Neither patient received trifluridine, though both were treated with levofloxacin and prednisolone eye drops for secondary bacterial conjunctivitis.

## Conclusions

Mpox can cause severe damage to multiple organs, including the eyes. The likelihood of death substantially increases in individuals co-infected with HIV, TB, or other sexually transmitted infections due to weakened immune systems. Early detection of mpox, particularly in high-risk populations, is vital for timely intervention and management. Supportive care remains the cornerstone of treatment, helping to improve outcomes and reduce complications. Therefore, prioritizing prompt diagnosis and comprehensive care is essential to enhance prognosis and reduce mpox-related mortality.
